# Genetic Diversity of Australian *Bacillus anthracis* Isolates Revealed by Multiple-Locus Variable-Number Tandem Repeat Analysis

**DOI:** 10.3390/microorganisms8060886

**Published:** 2020-06-11

**Authors:** Janine Muller, Ilhan Mohammad, Simone Warner, Roger Paskin, Fiona Constable, Mark Fegan

**Affiliations:** 1 Agriculture Victoria, Department of Jobs Precincts and Regions, Agribio, La Trobe University, 5 Ring Road, Bundoora, Victoria 3083, Australia; ilhan.mohammad@agriculture.vic.gov.au (I.M.); Fiona.constable@agriculture.vic.gov.au (F.C.); m_n.fegan@internode.on.net (M.F.); 2 Environment Protection Authority Victoria, Centre for Applied Sciences, Ernest Jones Drive, Macleod, Victoria 3085, Australia; simone.warner@epa.vic.gov.au; 3 OMNI Animal Health Consultancy, 6/35 McLaren Street, Mount Barker, South Australia 5251, Australia; rdpaskin@yahoo.com

**Keywords:** anthrax, *Bacillus anthracis*, genotyping, Multiple-Locus Variable-Number Tandem Repeat Analysis (MLVA)

## Abstract

Outbreaks of anthrax occur sporadically in Australia and most commonly in the “anthrax belt”, a region which extends from southern Queensland through the centre of New South Wales and into northern Victoria. Little is known about the epidemiological links between *Bacillus anthracis* isolates taken from different outbreaks and the diversity of strains within Australia. We used multiple-locus variable-number tandem repeat analysis employing 25 markers (MLVA25) to genotype 99 *B. anthracis* isolates from an archival collection of Australian isolates. MLVA25 genotyping revealed eight unique genotypes which clustered within the previously defined A3 genotype of *B. anthracis*. Genotyping of *B. anthracis* strains from outbreaks of disease in Victoria identified the presence of multiple genotypes associated with these outbreaks. The geographical distribution of genotypes within Australia suggests that a single genotype was introduced into the eastern states of Australia, followed by the spread and localised differentiation of the pathogen (MLVA25 genotypes MG1-MG6) throughout the anthrax belt. In contrast, unexplained occurrences of disease in areas outside of this anthrax belt which are associated with different genotypes, (MLVA25 genotypes MG7 and MG8) indicate separate introductions of *B. anthracis* into Australia.

## 1. Introduction

*Bacillus anthracis*, the causative agent of anthrax, is a Gram-positive spore-forming bacterium affecting livestock and humans worldwide. Anthrax is endemic in many areas of the world [1], including Australia, and can impact the international trade of livestock and their products. Outbreaks of anthrax have been recorded in Australia for over 170 years, with the first occurrence of the disease confirmed at Leppington in New South Wales in 1847 and the first reported outbreak in Victoria in 1876 [2]. More recently, outbreaks of anthrax in Australia have been largely confined to an area known as the “anthrax belt”, a region which extends from southern Queensland through the centre of New South Wales and into northern Victoria [3]. Sporadic occurrences of disease outside of this belt, as defined by Barro et al. [3], do occur but are considered rare. Two notable incidents occurred in areas geographically distinct from the historical anthrax belt. One was located in Queensland on the Marlborough Peninsula, over 750 km further north of the belt, and the second in Walpole, Western Australia, over 3000 km west of the belt [4]. Anthrax has not been reported from the Northern Territory, and no cases have been reported from South Australia or Tasmania since 1914 and 1933, respectively [4].

Multiple-locus variable-number tandem repeat analysis (MLVA) using a minimum of eight (MLVA8) [5] to a maximum of 31 [6] variable-number tandem repeat (VNTR) markers has been successfully employed to discriminate between *B. anthracis* isolates throughout the world [5,7,8,9,10,11,12,13]. The use of MLVA by Keim et al. [5,14] was central to the success of identifying different genetic clades, subgroups and strains of *B. anthracis*. VNTR markers are highly stable [15] and therefore reliable for differentiating closely related isolates. This reliability makes MLVA a valuable tool for use in understanding and interpreting the epidemiology of disease origins and outbreaks [15].

Thirty Australian *B. anthracis* isolates have been previously genotyped using the MLVA8 and MLVA15 typing schemes employed by Keim et al. [5] and Van Ert et al. [16]. These studies identified three different genotypes in circulation within Australia. However, the exact geographic location of the Australian genotypes used within the study was not defined, with the exception that most strains were sourced from an anthrax outbreak in Victoria in 1997.

The aim of this work was to build on this foundation by examining a broader representation of Australian *B. anthracis* isolates using the expanded MLVA25 typing scheme described by Lista et al. [17]. The MLVA25 technique was used in preference to the MLVA8 or MLVA15 typing schemes due to the greater discriminatory power of this method [15,16]. The diversity of 98 Australian *B. anthracis* isolates sourced from various geographic locations between 1993 and 2009 and one historical isolate from 1979 in New South Wales were examined. This report used historical epidemiological outbreak data in combination with highly discriminatory genotyping technologies to redefine our understanding of anthrax in Australia.

## 2. Materials and Methods

### 2.1. Bacillus anthracis Isolates

A total of 99 *B. anthracis* isolates from Australia, held at the National Anthrax Reference Laboratory, Department of Jobs, Precincts and Regions, Victoria, were used in this study. Isolates were confirmed as *B. anthracis* based on morphological characteristics when grown on sheep blood agar (SBA) and polymyxin, lysozyme, EDTA, thallous acetate (PLET) agar [18] at 37 °C for 24 and 48 h, respectively. All strains were further characterised by PCR amplification using the PCR methods described by Antwerpen et al. [19] and Berg et al. [20].

All isolates were from outbreaks of disease which occurred between 1993 and 2009, except for a single New South Wales isolate from 1979 (Table 1). Isolates were representative of the current geographic distribution of the disease in Australia and were collected from a range of sources (Table 1). Most isolates were from Victoria (73 isolates), but isolates from New South Wales (12 isolates), Queensland (11 isolates) and Western Australia (three isolates) were also genotyped. The 73 isolates from Victoria included 30 isolates from an outbreak of disease in 1997 and 28 isolates from a disease outbreak in 2007. Isolates were routinely cultured on SBA plates at 37 °C for 24 h.

### 2.2. DNA Isolation 

DNA was extracted using either a QIAamp DNA Mini Kit as per the manufacturer’s instructions (Qiagen, Chadstone, Victoria, Australia) or by the boiling of a cell suspension as described by Keim et al. [5]. Any spores contaminating the DNA extracts were removed using 0.1 µm Ultrafree-MC filter units (Merck Millipore, Bayswater Victoria, Australia) as described by Dauphin et al. [21], the sterility of extracts was confirmed by plating 20 µL of each extract onto an SBA plate followed by incubation at 37 °C overnight.

### 2.3. Multiple-Locus Variable-Number Tandem Repeat Analysis 

MLVA analysis using 25 loci was carried out as outlined by Lista et al. [17] and modified by Ted Hadfield, formerly of Midwest Research Institute, Florida (Pers. Comm.). Primers were labelled with one of four fluorescent dyes (6-carboxyfluorescein, VIC, NED or PET). PCR amplification of VNTR alleles was performed on a C1000 thermal cycler (Bio-Rad, Gladesville, New South Wales, Australia) using a Type-it Microsatellite PCR Kit (Qiagen, Chadstone, Victoria, Australia) as per the manufacturer’s instructions. A 2 µL aliquot of each amplification product was subjected to electrophoresis on a 2% agarose gel containing Invitrogen Sybr® Safe (Thermo Fisher Scientific, Scoresby, Victoria, Australia), and visualised by UV transillumination to ensure that the VNTR markers had amplified prior to separation by capillary electrophoresis on a 3730 × l DNA analyser at the Australian Genome Research Facility. Fragment sizes were estimated using GeneMapper v. 3.7 software by comparison to a GeneScan™ 1200 LIZ® Size Standard (Thermo Fisher Scientific, Scoresby, Victoria, Australia).

A representative of each VNTR allele size was reamplified using unlabelled primers, and amplicons were sequenced to confirm the amplicon size and VNTR repeat number using ABI Prism BigDye Terminator Sequencing at the Australian Genome Research Facility. An allele number string, based upon the number of tandem repeats present in each of the 25 VNTR markers, was assigned to each isolate (see Table 2).

### 2.4. Data Analysis 

The allele number string representative of each isolate was imported into Bionumerics v.6.1 (Applied Maths, Sint–Martens–Latem, Belgium) and compared to the publicly available database at MLVAbank for Microbe Genotyping (http://mlva.i2bc.paris–saclay.fr/) [22]. Data were analysed using categorical values and Unweighted Pair Group Method with Arithmetic Mean (UPGMA) analysis to generate trees from allelic profile data. Individual marker diversity within the population studied was assessed by determining Simpson’s index of diversity using the VNTR Diversity and Confidence Extractor (V–DICE) (Colindale, London, United Kingdom). The index of discrimination was calculated from the distribution of types with the Discriminatory Power Calculator (http://insilico.ehu.es/mini_tools/discriminatory_power/index.php).

## 3. Results

### 3.1. Marker Diversity

Only 12 of the 25 VNTR markers were informative for the population of *B. anthracis* isolates assessed in this study with diversity indices varying from 0 to 0.557 (Table 2). The VNTR marker with the greatest discriminatory power was the Bams22 marker (DI = 0.557) which differentiated three alleles within the 99 isolates.

### 3.2. Genetic Diversity of Bacillus anthracis Strains in Australia Using MLVA25

**Figure 1:**
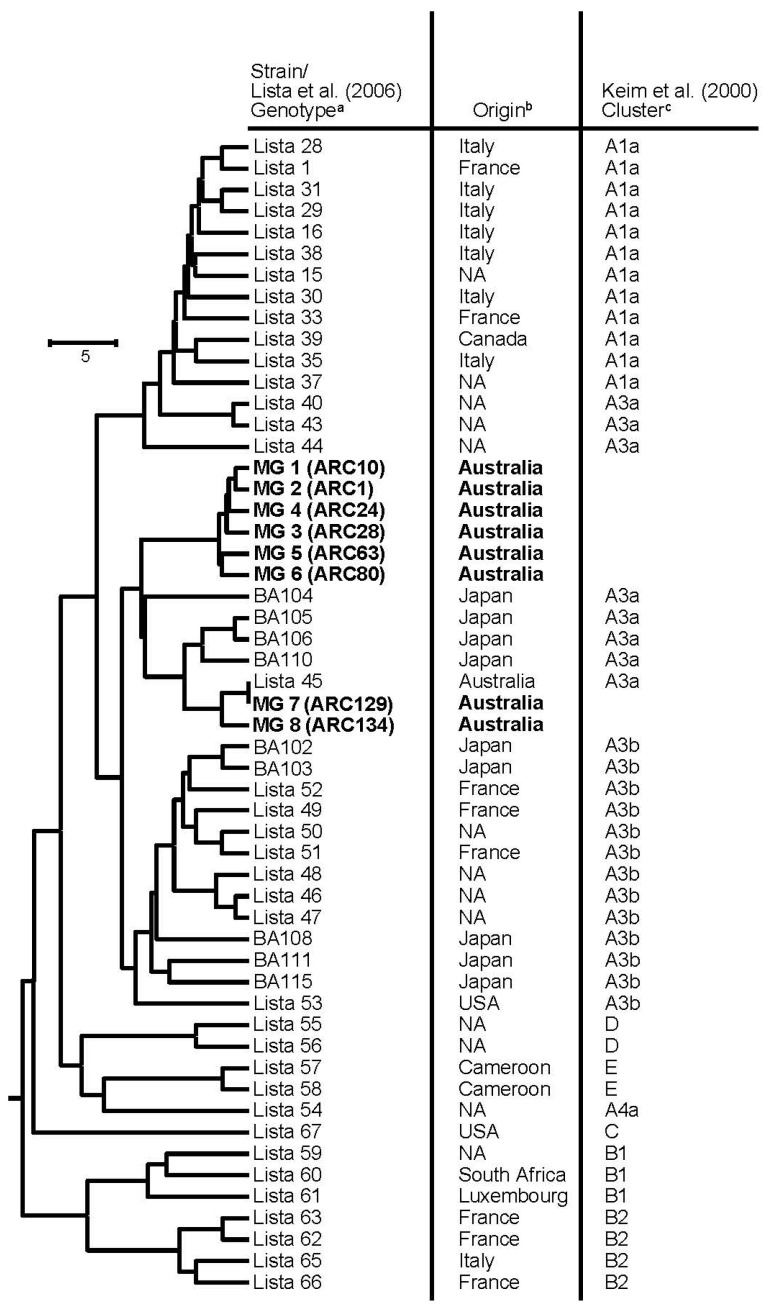
Phylogenetic relationship of *B. anthracis* strains, including Australian strains (bold type) assessed by MLVA25 genotyping. ^a^ Strains prefixed by “Lista” refer to the MLVA25 genotypes described by Lista et al. [17]. Strains prefixed by “BA” are strains representing MLVA25 genotypes described by Okutani et al. [23]. Strains prefixed MG are strains representing genotypes described in this study; ^b^ Country of origin of strains where known, NA indicates that the origin is unknown; ^c^ Cluster of strains as defined by Keim et al. [5] and adapted by Lista et al. [17] and Okutani et al. [23].

The 99 Australian *B. anthracis* strains fell into eight distinct MLVA25 genotype patterns, MG 1–MG 8 (Figure 1, Table 1). All eight MLVA patterns were related to strains within the previously defined A3 genotype based upon the MLVA8 typing system [5,17] (Figure 1). Most of the strains (94 of 99; Table 1) belonged to six (MG 1–MG 6) of the eight genotypes and formed a monophyletic cluster (Figure 1). The remaining five strains fell into two related genotypes (MG 7 and MG 8), which clustered with a previously genotyped strain from Australia (Lista 45; Figure 1). A total of 78 strains fell into the MLVA genotypes MG 1 and MG 2 (Table 1). Genotype MG 1 contained 31 Victorian isolates from 1997, 2003, 2005 and 2007 (Table 1). Genotype MG 2 contained 47 strains taken from Victoria, New South Wales and southern Queensland (Table 1). Isolates in MG 3 and MG 4 were isolated from Victoria during 1997, 2004 and 2008. The single strain comprising genotype MG 5 was isolated from southern Queensland in 2002, and the four strains in genotype MG 6 were isolated from New South Wales in 1979 and 2007/2008. The three strains in genotype MG 7 were isolated from Walpole in Western Australia, and the two strains in MG 8 were isolated from a disease outbreak on the Marlborough Peninsula, north of Rockhampton in Queensland. 

### 3.3. Global Comparison of Strains Using MLVA

Examination of global strains shows that the strains most closely related to Australian *B. anthracis* types were collected in Japan (Figure 1).

### 3.4. Epidemiologically–Linked Isolates

Analysis of epidemiologically–linked isolates from outbreaks throughout Australia revealed more than one genotype might have been responsible for disease outbreaks. The 30 strains analysed from the Victorian anthrax outbreak in 1997 included four genotypes; MG 1 (24 isolates), MG 2 (three isolates), MG 3 (two isolates) and MG 4 (one isolate) (Table 1). The 28 strains taken from the Victorian outbreak in 2007 included two genotypes, MG 1 (four isolates) and MG 2 (24 isolates) (Table 1). Isolates taken from a further Victorian anthrax outbreak in 2004 included two genotypes, MG 1 (one isolate) and MG 3 (six isolates). Isolates taken from an outbreak of anthrax in Queensland in 2002 included two genotypes, MG 2 (eight isolates) and MG 5 (one isolate). All other epidemiologically linked isolates from outbreaks of disease fell within a single genotype.

## 4. Discussion

The 99 Australian isolates of *B. anthracis* genotyped in this study represent eight distinct genotypes in two clusters based upon MLVA25 analysis. All strains fall within the A3 cluster described by Keim et al. [5], which has been termed the most important group of *B. anthracis* strains due to its wide distribution and prevalence throughout the world [5].

The discriminatory power of any molecular typing system is generally improved by increasing the number of loci assessed [14,17]. Hence, the MLVA25 method was employed in this study. Previous studies by Keim et al. and Van Ert et al., which included a set of Australian strains of *B. anthracis*, employed MLVA8 [5] and MLVA15 [16]. Results of MLVA8 analysis performed by Keim et al. on 30 Australian strains [5] revealed the presence of three genotypes (designated genotypes 54, 55 and 66). As the MLVA25 typing scheme includes the eight markers used by Keim et al., it was possible to show that by restricting the analysis to the MLVA8 markers only, four genotypes were recognised within the 99 isolates tested in the present study, whereas eight were recognised using MLVA25 (results not shown). From their genotyping data using MLVA15, Van Ert et al. [16] concluded that there had been separate introductions of *B. anthracis* into Australia. Our genotyping data, together with data on the geographic origin of strains within Australia, also indicate that this is likely. Strains present in the eastern states of Australia primarily belong to a monophyletic group of strains (MG 1–MG 6), whereas strains isolated from Western Australia (MG 7) and the Marlborough Peninsula in Queensland (MG 8) belong to a separate cluster of Australian strains.

After the introduction of anthrax into the eastern states of Australia in the late 1840s, probably in contaminated bone meal, anthrax spread along stock routes throughout New South Wales and southern Queensland and was later introduced into Victoria in 1876 [2]. In contrast, the first and only outbreak of anthrax in Western Australia occurred on three farms around Walpole in the south of the state in 1994 [24], and the disease has not been found in Western Australia since this time. Similarly, in 1993 on the Marlborough Peninsula in Queensland, anthrax was identified on a single farm, and the disease has not been found in this region since. The Marlborough Peninsula is geographically distant from the areas of historical outbreaks of disease in Queensland, which are found in the south of the state close to the border with New South Wales [2]. The sources of these outbreaks of disease in Western Australia and the Marlborough Peninsula in Queensland are unknown [24,25]. The differences in MLVA25 genotypes between strains isolated from the anthrax belt and the Western Australian and Marlborough Peninsula outbreaks, together with the geographic separation of these outbreaks, indicates that there have been at least two separate introductions of *B. anthracis* into Australia.

The most commonly isolated and widely distributed genotype identified within this study was MG 2. This genotype was found throughout the anthrax belt spanning southern Queensland, New South Wales and northern Victoria. Isolates belonging to the closely related genotypes MG 1, MG 3 and MG 4 were only found in Victoria. All four of these genotypes (MG 1–MG 4) were found in the collection of isolates from the large 1997 anthrax outbreak in the Goulburn Valley area of northern Victoria. This disease outbreak occurred in a relatively small but intensively dairy–farmed area where anthrax had not been seen since 1914 [26]. The four genotypes associated with the outbreak differed from one another in the length of only one or two alleles over the 25 alleles that were assessed (Table 2). It has been reported that MLVA alleles of *B. anthracis* isolates are relatively stable over time, with few mutations observed over many passages of *B. anthracis* strains [5]. According to Keim et al. [5], the available evidence suggests that VNTR mutation rates are slower than 10^–5^ and that mutational changes are thought to occur in single–repeat steps. It is therefore unlikely that these genotypes originated spontaneously during the outbreak, and consequently, multiple genotypes gave rise to concurrent parallel single outbreaks of disease. Similarly, smaller outbreaks of disease in the same geographic area in 2004 and 2007 were associated with two of the four genotypes (MG 2 and MG 3 in 2004; MG 1 and MG 2 in 2007). Smith et al. [8] have also identified the existence of multiple genotypes in outbreaks of disease in Kruger National Park. However, these genotypes rarely overlapped in their geographic location within the park in the same outbreak year [7] unlike the situation in the Victorian disease outbreaks. In contrast, other researchers have determined that isolates from outbreaks of disease belong to a single MLVA type. Kenefic et al. [27] employed MLVA15 genotyping on 47 strains from an outbreak of disease in South Dakota and identified that only a single genotype was present. Similarly, Garofolo et al. [28] studied 53 strains from an outbreak in southern Italy using MLVA25 genotyping and found only a single genotype to be present. More recently, Rondinone et al. [11] employed MLVA31 to study 234 strains across Italy and showed that most genotypes were exclusive to each region, demonstrating the highly indigenous nature of the Italian strains. Given that multiple MLVA25 genotypes are responsible for a single outbreak of disease in Victoria, our results substantiate the suggestion that environmental conditions lead to the emergence of *B. anthracis* from soil reservoirs to cause disease outbreaks and that outbreaks are not only the result of animal to animal transmission from a single point source [14].

The greatest diversity of strains was identified in *B. anthracis* isolates from the state of Victoria, but this could be expected as most genotyped strains in this study were isolated from this state. Low sample numbers from New South Wales and southern Queensland could have resulted in an underestimation of the diversity of *B. anthracis* in these regions. It may be that there is greater diversity in these regions and amongst the isolates that were collected, but the discriminatory power of MLVA25 was not great enough to reveal it. Given that anthrax was first identified in New South Wales, and that outbreaks of disease have continued to occur over a large geographic area since this time, it was expected that the diversity of strains from New South Wales would have exhibited the greatest diversity. 

In New South Wales, anthrax occurs most commonly in the anthrax belt that runs through the centre of the state [2,29]. However, in December 2007 through to January 2008, an outbreak of disease occurred in the Hunter Valley region of New South Wales, an area which was historically considered to be outside of the anthrax belt [29]. The redefining of the anthrax belt by Barro et al. [3] has expanded the area thought to support the survival of anthrax spores and this region now sits within the newly defined belt. Anthrax had not been described in this area since 1939 [2,29]. The three strains that were genotyped from this outbreak belonged to genotype MG 6, which also contained an isolate of unknown origin within New South Wales from 1979. Genotype MG 6 differs from the genotype commonly found in New South Wales (MG 2) by a single marker (Table 2). It may be speculated that this genotype is unique to this area of New South Wales. Irrespective of the geographic distribution of this genotype, it is probable that this outbreak did not originate from the importation of contaminated animals or feed, and the hypothesis of Durrheim et al. [23] that climatic conditions and soil disturbance may have exposed historic anthrax spores from 1939 is most likely.

Strains belonging to genotype MG 7 from Western Australia have the same MLVA25 profile as the Lista 45 genotype [17], which is equivalent to the MLVA8 genotype 55 of Keim et al. [5]. This genotype (Lista 45) represents the pattern expected from the strain Australia94, for which a genome sequence is available. The exact origin of this strain is confused within the available scientific literature. This strain is equivalent to the strain 29/32 from the Centre for Applied Microbiology and Research, Porton Down, United Kingdom, and K4834 [5] and strain A0039 [16]. Previous publications have indicated that this strain, and therefore by extension, this genotype, originated in Victoria [30,31] This does not match the geographic distribution of this genotype in the present study, as the isolates with this genotype were found only in Western Australia. However, tracing the origin of this strain has revealed that the strain does originate from Western Australia (Martin Hugh–Jones, Louisiana State University and Peter Turnbull, Pers. Comm.). Therefore, the strain Australia94 (29/32, K4834, A0039 and MG 7) originated from Western Australia as would be expected for a strain of this genotype.

## 5. Conclusions

This work employed the MLVA25 genotyping method to extend the number of *B. anthracis* genotypes known to be present in Australia. A baseline of the genetic diversity of *B. anthracis* strains found in Australia was established that could be used for future epidemiological and forensic analyses, allowing the identification of any further introductions of *B. anthracis* into Australia.

The use of MLVA25 has revealed that *B. anthracis* strains from the 1997, 2004 and 2007 outbreaks of anthrax in Victoria, Australia, are more diverse than previously thought, with multiple genotypes being recognised within this group of strains. However, the overall genetic diversity of *B. anthracis* within Australian isolates is relatively low and the geographic distribution of related genotypes indicates that this genotype was spread throughout the anthrax belt along stock routes, following the introduction of a single genotype into New South Wales in the mid–1840s, as hypothesised by Seddon and Albiston [2]. The spread of the pathogen was then followed by limited localised differentiation into other closely related genotypes. However, unexplained occurrences of disease in areas outside of this anthrax belt, which are associated with different genotypes, indicates that separate introductions of *B. anthracis* into Australia have occurred.

Future work will employ whole genome sequence analysis to further improve the resolution of the genomic diversity of *B. anthracis* isolates from Australia. Whole genome sequencing is a powerful alternative method to MLVA, with the versatility to look at disease outbreak origins, introductions, and routine surveillance to support epidemiological investigations. This technology is becoming standard practice for examining the genetic diversity of clonal bacterial species such as *B. anthracis* [32,33]. Rapid classification frameworks made available by researchers such as Bruce et al. [32] offer a simplified and robust tool for the analysis of *B. anthracis* evolution and classification. Owing to the expansion of available genome sequence data and the comparison of *B. anthracis* strains worldwide [32,33,34,35,36,37], this approach offers greater insight into the global placement of the Australian strains and may provide a deeper understanding of the genomic diversity at a local level.

## Figures and Tables

**Table 1:** Information on strains used in this study.

MLVA Genotype	Geographic Iocation State (Town or Area)	Dates Isolated	Number of Strains	Sources
MG 1	Victoria (Tatura)	1997	24	Ovine, Bovine, Soil/Water, Flies
	Victoria (Tatura)	2003	1	Bovine
	Victoria (Tatura)	2005	1	Bovine
	Victoria (Tatura, Stanhope)	2007	4	Bovine
	Victoria (Kyabram)	2009	1	Bovine
MG 2	Victoria (Tatura)	1997	3	Flies, Ovine, Soil
	Victoria (Swan Hill)	2002	1	Bovine
	Victoria (Harston)	2004	1	Bovine
	Victoria (Stanhope, Wyuna)	2007	24	Bovine, Soil
	Victoria (Tatura)	2008	1	Bovine
	Victoria (Stanhope)	2009	1	Bovine
	NSW (Narrandera)	2002	1	Ovine
	NSW (Armidale) *	1994	1	Bovine
	NSW (Deniliquin)	2003	1	Bovine
	NSW (Narrandera)	2004	2	Ovine
	NSW (Yenda, Berrigan)	2005	3	Ovine, Bovine
	Qld (Wandoan, Dirranbandi)	2002	8	Bovine
MG 3	Victoria (Tatura)	1997	2	Bovine, Flies
	Victoria (Harston)	2004	6	Bovine
	Victoria (Harston)	2008	2	Bovine
MG 4	Victoria (Tatura)	1997	1	Bovine
MG 5	Qld (Dirranbandi)	2002	1	Bovine
MG 6	NSW (Armidale) *	1979	1	Bovine
	NSW (Hunter Valley)	2007/2008	3	Bovine
MG 7	WA (Walpole)	1994	3	Bovine
MG 8	Qld (Rockhampton)	1993	2	Bovine

* The exact geographic origin of the strain from “Armidale” in New South Wales (NSW) is unknown.

**Table 2:** Multilocus genotypes of Australian *Bacillus anthracis* isolates and diversity indices for each marker locus for the *B. anthracis* isolates typed.

Marker Loci	MLVA Genotype ^a^								Diversity Index ^b^	Confidence Interval	K	max (pi)
	MG 1	MG 2	MG 3	MG 4	MG 5	MG 6	MG 7	MG 8				
CG3	2	2	2	2	2	2	2	2	0	0.000–0.069	1	1
Bams44	8	8	8	8	8	8	8	8	0	0.000–0.069	1	1
Bams3	30	30	30	30	30	30	30	30	0	0.000–0.069	1	1
VrrB2	7	7	7	7	5	7	7	7	0.02	0.000–0.057	2	0.99
Bams5	6	6	6	6	6	6	7	7	0.129	0.044–0.214	2	0.931
Bams15	45	45	45	45	45	45	45	45	0	0.000–0.069	1	1
Bams1	16	16	16	16	16	16	16	16	0	0.000–0.069	1	1
VrrC1	33	33	33	33	33	33	57	57	0.129	0.044–0.214	2	0.931
Bams13	70	70	70	70	70	70	73	70	0.094	0.018–0.170	2	0.95
VrrB1	16	16	16	16	16	16	12	12	0.129	0.044–0.214	2	0.931
Bams28	14	14	14	14	14	14	14	14	0	0.000–0.069	1	1
VrrC2	17	17	17	17	17	17	17	17	0	0.000–0.069	1	1
Bams53	8	8	8	8	8	8	8	8	0	0.000–0.069	1	1
Bams31	64	64	64	40	64	64	64	64	0.02	0.000–0.057	2	0.99
VrrA	10	10	10	10	10	10	10	10	0	0.000–0.069	1	1
Bams25	13	13	13	13	13	13	13	13	0	0.000–0.069	1	1
Bams21	10	10	10	10	10	10	9	9	0.129	0.044–0.214	2	0.931
Bams34	8	8	8	8	8	8	8	8	0	0.000–0.069	1	1
Bams24	11	11	11	11	11	11	11	11	0	0.000–0.069	1	1
Bams51	9	9	7	9	9	9	9	9	0.178	0.085–0.272	2	0.901
Bams22	17	16	17	17	16	16	13	13	0.557	0.513–0.601	3	0.515
Bams23	11	11	11	11	11	11	11	11	0	0.000–0.069	1	1
Bams30	57	57	57	57	57	57	54	57	0.094	0.018–0.170	2	0.95
pX01	6	6	6	6	6	6	8	8	0.129	0.044–0.214	2	0.931
pX02	8	8	8	8	8	9	9	9	0.194	0.099–0.289	2	0.891

^a^ Different alleles for each marker, using multiple–locus variable–number tandem repeat analysis (MLVA) genotype MG 1 as a reference, are indicated by grey shading. ^b^ Diversity is based upon Simpson’s index, calculated using all 99 isolates. The greater the value, the greater the sample diversity.
